# Clinical relevance of Neutral Endopeptidase (NEP/CD10) in melanoma

**DOI:** 10.1186/1479-5876-5-2

**Published:** 2007-01-05

**Authors:** Elsa F Velazquez, Molly Yancovitz, Anna Pavlick, Russell Berman, Richard Shapiro, Dusan Bogunovic, David O'Neill, Yi-Lo Yu, Joanna Spira, Paul J Christos, Xi Kathy Zhou, Madhu Mazumdar, David M Nanus, Leonard Liebes, Nina Bhardwaj, David Polsky, Iman Osman

**Affiliations:** 1 Ronald O. Perelman Department of Dermatology, New York University School of Medicine, New York, NY, USA; 2 Department of Medicine, New York University School of Medicine, New York, NY, USA; 3 Department of Surgery, New York University School of Medicine, New York, NY, USA; 4 Department of Public Health, Weill Medical College of Cornell University, New York, NY, USA; 5 Department of Medicine, Weill Medical College of Cornell University, New York, NY, USA; 6 Department of Pathology, Brigham and Women's Hospital, Harvard School of Medicine, Boston, MA, USA

## Abstract

**Background:**

Overexpression of Neutral Endopeptidase (NEP) has been reported in metastatic carcinomas, implicating NEP in tumor progression and suggesting a role for NEP inhibitors in its treatment. We investigated the role of NEP expression in the clinical progression of cutaneous melanoma.

**Methods:**

We screened 7 melanoma cell lines for NEP protein expression. NEP-specific siRNA was transfected into the lines to examine the role of gene transcription in NEP expression. Immunohistochemistry was done for 93 specimens and correlated with clinicopathologic parameters. Thirty-seven metastatic melanoma specimens were examined for NEP transcript expression using Affymetrix GeneChips. In a subset of 25 specimens for which both transcript and protein expression was available, expression ratios were used to identify genes that co-express with NEP in GeneChip analysis.

**Results:**

NEP was overexpressed in 4/7 human melanoma cell lines, and siRNA knock-down of NEP transcripts led to downregulation of its protein expression. NEP protein overexpression was significantly more common in metastatic versus primary tumors (P = 0.002). Twelve of 37 (32%) metastatic tumors had increased NEP transcript expression, and an association was observed between NEP transcript upregulation and protein overexpression (P < 0.0001). Thirty-eight genes were found to significantly co-express with NEP (p < 0.005). Thirty-three genes positively correlated with NEP, including genes involved in the MAP kinase pathway, antigen processing and presentation, apoptosis, and WNT signaling pathway, and 5 genes negatively correlated with NEP, including genes of focal adhesion and the notch signaling pathways.

**Conclusion:**

NEP overexpression, which seems to be largely driven by increased transcription, is rare in primary melanoma and occurs late in melanoma progression. Functional studies are needed to better understand the mechanisms of NEP regulation in melanoma.

## Background

Neutral endopeptidase (NEP, also known as CD10, MME, CALLA) is a 90–100 kDA cell surface peptidase that inactivates a variety of physiologically active peptides. Altered NEP expression has been shown to play a role in many non-neoplastic [1-3] and neoplastic disaeases [4-8]. In many tumors, such as prostate and small-cell lung cancer, NEP is thought to act as a tumor suppressor, as its expression is down-regulated with tumor progression [4,5]. In this regard, we have previously shown that loss of NEP in cultured prostate cancer cells stimulates cell proliferation and migration [9]. We also demonstrated that complete loss of NEP expression was independently associated with prostate cancer recurrence after surgery [6].

Data from other tumor types, however, reveal a more complex role of NEP in neoplastic disorders. Several independent studies have shown a correlation between increased NEP, rather than decreased or absent expression, and tumor progression [7,8,10]. An association between NEP expression and increased proliferation was reported in aggressive non-Hodgkin lymphoma [8], and increased NEP expression has been shown to correlate with invasion and liver metastasis in colorectal carcinoma [7,10]. Other investigators have demonstrated that tumor-specific expression of NEP in stromal cells may facilitate invasion and metastatic progression in gastric, breast and colorectal carcinomas [11-13].

The association between increased NEP expression and melanoma progression is of particular therapeutic interest given the availability of NEP inhibitors [14,15]. In our study, we screened several melanoma cell lines for NEP protein expression and examined increased transcription as a possible mechanism of its protein overexpression. We further examined NEP transcription and protein expression in a well-characterized cohort of melanoma patients. We then explored the Genechip data to determine if there were other genes whose expression correlated with NEP expression. Both our *in-vitro *and *in-vivo *data suggest that NEP overexpression is largely driven by increased transcription. We also demonstrate that NEP overexpression is a rare event in primary melanoma and occurs more commonly in metastatic melanoma. NEP overexpression did not seem to have a strong prognostic value in our study cohort. Functional studies are underway to determine the mechanisms of NEP regulation in melanoma.

## Methods

### Protein extraction, immunoprecipitation, and Western blot analysis

Seven human metastatic melanoma cell lines were studied, including SK-MEL-19, -23, -29, -85, -100, -197 (gifts of Dr. Alan Houghton) and Mewo (American Type Culture Collection, Manassas, VA). The SK-MEL cell lines were cultured in Dulbecco's modified Eagle's medium supplemented with 10% fetal bovine serum, 2 mM L-glutamine and 1% penicillin/streptomycin. The Mewo cell line was maintained in modified Eagle's medium containing 10% Fetal Bovine Serum. All cell lines were routinely grown at 37°C under 5% CO2. The cells were passed two times weekly in order to keep them in the exponential growth phase. Cells were washed with cold PBS and then lysed with an ice-cold buffer (pH 7.0) containing 10 mM Tris-HCl (pH 7.5), 1 mM EDTA, 400 mM NaCl, 10% glycerol, 0.5% NP40, and protease and phosphatase inhibitors. Lysates were placed on ice for 20 minutes before clarification by centrifugation. Protein determinations were performed using the Bradford method (Bio-Rad Laboratories, Hercules, CA). Twenty-five to 50 μg of each sample were fractionated by SDS-PAGE and transferred to Immobilon-P membranes (Millipore, Bedford, MA). Membranes were blocked with 8% nonfat dry milk, 0.1% Tween 20 in PBS, and probed with the anti-NEP mouse monoclonal antibody, NCL-CD-10-270 (1:100, NovaCastra Laboratories Ltd., Newcastle upon Tyne, UK). Protein loading was confirmed using the goat anti-Ran monoclonal antibody (SC-1156, 1:200, Santa Cruz Biotechnology). Bands were visualized using the following horseradish peroxidase-conjugated secondary antibodies: anti-mouse (SC-2055, 1:2000, Santa Cruz Biotechnology), and anti-goat (SC-2020, 1:4,000, Santa Cruz Biotechnology) and the SuperSignal West Pico chemiluminescent substrate (Pierce, Rockford, IL).

### NEP siRNA transfection

Two million cells were transfected with 10 ug of siRNA (Smartpool from Dharmacon Research, Inc., Lafayette, CO) directed against NEP, or control non-specific siRNA using the Amaxa Nucleofector system. Program U-20 was selected for the SK-MEL-19 cell line and A-23 for the Mewo cell line. Buffers used for the Nucleofecter were NHEM solution for SK-MEL-19 and V solution for Mewo. Post-transfected cells were transferred to 0.5 ml pre-warmed RPMI1640 medium for 10 minutes at 37°C in a CO_2 _incubator, and then seeded at 3.2 × 10^5^/well in 24 well plates. Cells were incubated in a humidified 37°C CO_2 _incubator for 48 hours. For each cell line, triplicate transfections were conducted on the same day.

### Patient characteristics

The study cohort consisted of 84 melanoma patients identified through the Interdisciplinary Melanoma Cooperative Group database at the New York University (NYU) School of Medicine (39 females, 45 males, mean age 58.5 ± 18.1 years). Ninety-three specimens from 84 patients were used for immunohistochemistry, and 37 specimens from 32 patients were used for transcript expression analysis. Of these, 25 specimens from 22 patients were analyzed by both methodologies based on corresponding paraffin tissue availability. The Interdisciplinary Melanoma Cooperative Group has been enrolling patients since September 2002. Those patients with either follow-up of at least 2 years and/or available fresh tissue samples for microarray gene expression analysis were selected for this study. The study was approved by the NYU Institutional Review Board and all patients signed informed consent before enrollment. Relevant clinicopathologic, demographic and survival data were recorded for all patients.

### Microarray gene expression analysis

NEP transcript expression was assessed using Affymetrix U133Plus2.0 GeneChips on all available fresh tumor tissue specimens, which included 37 metastatic melanoma specimens obtained from 32 patients (3 patients had 2 metastases and 1 patient had 3 metastases included). Tissue was collected at time of surgery for metastatic disease, and placed in at least 10 volumes of RNAlater (Qiagen) at 4°C overnight, then stored at -80°C. Whole RNA was extracted from the tissue using the RNeasy Mini Kit from Qiagen. RNA was quantified by spectrophotometric assessments at 260/280 nm, and RNA quality was assessed by RNA 6000 Nano Assay using an Agilent 2100 Bioanalyzer (Agilent Technologies). Double stranded cDNA synthesis was performed using a SuperScript double-stranded cDNA synthesis kit from Invitrogen. *In-vitro *transcription of biotin-labeled cRNA probes was done using an IVT labeling kit (Affymetrix) following the kit's instructions. The cRNA probes were chemically fragmented using a fragmentation buffer (Affymetrix), and fragmented biotin-labeled cRNA was hybridized on Affymetrix Human Genome U 133 Plus 2.0 chips, in the Rockefeller University Genomics Core lab. The raw microarray data files were analyzed using BioConductor [16] packages under R [17]. Data were normalized using the robust multi-array average (RMA)[18] algorithm. Gene expression ratios between each tumor specimen and the lymph node control were calculated. Ratios greater than 2 were considered over expressed, otherwise they were considered as normal/under-expressed.

NEP transcript expression was analyzed in metastatic melanoma samples according to the absolute call values assigned by the chip reader, as well as in comparison to normal lymph node tissue. The chip reader cutoff corresponded to an intensity of 110–130, and the intensity of normal lymph node tissue for NEP transcription was 338. We thus used normal lymph node for comparison as a more stringent control, since the germinal proliferating centers of normal lymph node tissue displayed NEP expression on immunohistochemistry and the majority of the metastatic specimens studied were lymph node tissues. The use of normal lymph node tissue as a control in a similar setting was recently described by another group [19]. Increased NEP transcript levels were defined as greater than 2-fold increase of the absolute expression compared to NEP transcript expression in normal lymph node tissue. Comparison of NEP transcript regulation between metastatic melanoma and primary melanoma tissues was not feasible, since at our institution, primary melanomas are not available as frozen specimens; instead they are all formalin fixed after removal for diagnostic purposes.

For the 25 specimens for which we had both transcript and protein expression data, we identifed genes/probes that co-express with NEP by ranking each probe through multiple criteria using their expression ratios. First, we calculated the correlation of expression ratios between each probe and NEP using Pearson correlation coefficient, where NEP expression was defined as the average of two NEP probes that gave similar results. Secondly, the expression ratios were dichotomized based on the over-expression criteria. The correlation of the dichotomized expressions between each gene and the NEP was calculated using Kendall's method. Adding this criterion allowed for down-weighting genes that have similar expression pattern as NEP but are largely over-expressed or normal/under-expressed based on our over-expression criteria. Thirdly, we compared the expressions between the NEP over-expressed and NEP normal/under-expressed subjects for each gene using Wilcoxon rank sum test. Overall ranks of the genes were calculated by summing up the ranks based on the two correlations and the p-values from the Wilcoxon rank sum test. To ensure the expressions between the NEP over-expressed and NEP normal/under-expressed subjects differ and have relatively small variation, we also employed the criteria that the p-value from the t-test need to be less than 0.005.

### Immunohistochemical analysis and scoring

Ninety-three specimens from 84 patients were examined for NEP protein expression using an immunohistochemical assay, including 33 specimens from patients with Stage I/II disease, and 60 specimens from patients with Stage III/IV disease. Two patients had two primary melanoma tumors included in the analysis, five patients had two metastatic tumors included in the analysis, and one patient had three metastatic tumors included in the analysis. All tissue sections were formalin fixed and paraffin embedded. Expression of NEP was assessed using NCL-CD-10-270 (NovaCastra Laboratories Ltd., Newcastle upon Tyne, UK) at 1:25 dilution [20]. All immunohistochemistry was performed on an automated immunostainer (NexEs, Ventana Medical Systems) for standardization of the assay and to minimize experimental variation in results. An antigen retrieval protocol for enhancement of potentially masked epitopes was used. Sections were immersed in boiling 0.01% citric acid (pH 6.0) for twenty minutes under microwave treatment to enhance antigen retrieval, allowed to cool, and incubated with the NEP antibody overnight. Kidney sections demonstrating strong NEP expression were used as positive controls. Expression of NEP was scored by an attending pathologist (E.V.), who was blinded to the patients' clinical data. Scoring was based on the proportion of cells with positive surface membrane and cytoplasmic staining. Immunoreactivity was assessed on a continuous scale with values ranging from undetectable levels (0%) to homogenous staining (100%) of invasive melanoma cells. We then classified tumors into three groups: focal (undetectable-20%), moderate (20–60%) and diffuse (60–100%). Moderate or diffuse expression was considered overexpression based on the strong association between increased transcription and >20% (moderate and diffuse) NEP protein expression. This molecular association set >20% as the cut point for NEP protein overexpression.

Descriptive statistics were calculated for baseline demographic and clinicopathologic characteristics. Associations between NEP immunoreactivity and clinicopathologic features were assessed by Fisher's exact test, the chi-square test, or the Cochran-Armitage trend test, as appropriate. Three survival outcome measures, including overall survival (time from initial diagnosis of melanoma to death), disease-free survival (time from initial diagnosis of melanoma to first recurrence), and survival after first recurrence (time from first recurrence to death), were analyzed using the Kaplan-Meier method with the log-rank test employed to evaluate associations between NEP immunoreactivity status (overexpression versus no overexpression) and survival outcome measures. Median follow-up time was computed as the median survival time of the alive patients. All p-values are two-sided with statistical significance evaluated at the 0.05 alpha level. Ninety-five percent confidence intervals (95% CI) were calculated to assess the precision of the obtained estimates. All analyses were performed in SAS Version 9.1 (SAS Institute, Inc., Cary, North Carolina) and Stata Version 8.0 (Stata Corporation, College Station, Texas).

## Results

### NEP expression in cell lines and effect of NEP downregulation

**Figure 1:**
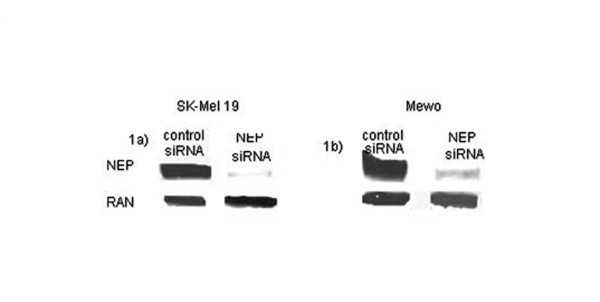
**NEP transcript downregulation in melanoma cell lines**. Melanoma SK-MEL 19 (**1a**) and MeWo (**1b**) cell lines were transfected with 10 ug of control siRNA (left column) and NEP siRNA (right column) in one million cells. The cells were harvested 48 hours post-transfection. Following siRNA transfection, there was decreased expression of NEP in both cell lines. RAN served as a control.

We analyzed in-vitro NEP expression using 7 melanoma cell lines. Four of 7 (57%) of the melanoma cell lines overexpressed NEP with the maximum level of expression observed in the MeWo and SK-MEL-19 cell lines. To investigate the role of gene transcription in NEP expression, NEP specific siRNA was transfected into the SK-MEL-19 and MeWo cell lines. This led to a marked reduction in NEP protein in the 2 cell lines tested (Figure 1).

### NEP protein expression in primary melanoma

NEP protein expression was examined in 33 primary melanoma tumor specimens from 31 patients with Stage I/II disease. The median tumor thickness for primary tumors was 1.0 mm (range 0.3–11.0 mm); with 17 tumors ≤ 1.0 mm, 13 tumors 1.01–4.0 mm, and 3 tumors >4.0 mm in Breslow thickness. Of the 31 patients whose primary melanomas were analyzed, 19 (61%) were Stage I and 12 (39%) were Stage II, according to the 6^th ^Edition of the AJCC staging guidelines. Twenty (61%) tumors were axial and 13 (39%) were on the extremities. Histologic examination revealed 23 (70%) superficial spreading type melanomas. The remainder were nodular (n = 5), desmoplastic (n = 2), acral, lentigo maligna and indeterminate (n = 1 for each). Only 1 of the 33 tumors diffusely overexpressed NEP. This was an 11.0 mm ulcerated acral melanoma detected in a 69 year-old Caucasian man who subsequently developed lymph node and liver metastases before dying of disease three years after diagnosis. The other primary melanomas were *completely negative *for NEP expression.

### NEP protein expression in metastatic melanoma

**Figure 2:**
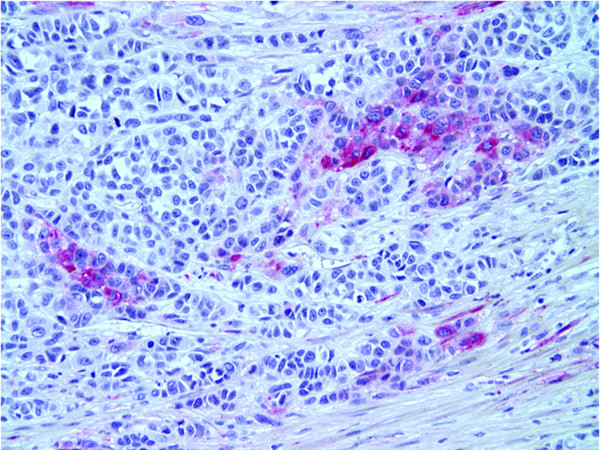
**Immunohistochemical detection of NEP expression in melanoma**. Metastatic melanoma showing focal expression of NEP (red stain) (20×).

**Figure 3:**
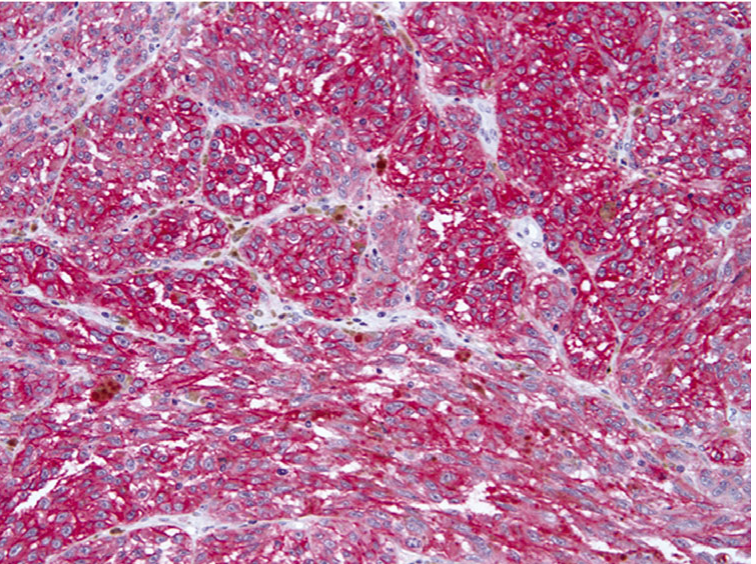
**NEP expression in melanoma by immunohistochemistry**. Metastatic melanoma showing diffuse NEP expression (red stain) (20×).

We next examined NEP expression in 60 melanoma tumors from 53 patients with Stage III/IV disease (27 lymph node metastases, 19 skin or subcutaneous metastases, 8 visceral metastases and 6 biopsies from primary lesions in patients with Stage III disease). Eighteen of the 60 (30%) specimens showed overexpression of NEP, which was significantly different compared to the primary tumor rate of 1/33 (3%) (P = 0.002 by Fisher's exact test). Table 1 displays the distribution of cases tested by immunohistochemistry. Figures 2 and 3 illustrate representative melanoma cases showing focal NEP expression (Figure 2), and diffuse NEP overexpression (Figure 3). There was a trend towards increased NEP overexpression in visceral metastases versus skin and lymph node metastases (57% (4/7) of patients with visceral metastases, versus 40% (6/15) and 24% (6/25) of patients with skin and lymph node metastases, respectively, p = 0.08 by trend test). Clinicopathologic correlation with NEP overexpression in metastatic melanoma samples revealed no significant association between increased NEP expression and age (27% of subjects ≤ 65 years-old overexpressed NEP versus 40% of subjects >65 years-old, p = 0.34) or gender (40% of females versus 25% of males overexpressed NEP, p = 0.24).

**Table 1:** NEP Protein Expression in Melanoma Specimens Tested by Immunohistochemistry

**Disease Stage:**	**Specimens tested (n)**	**NEP overexpression (n)**	**NEP overexpression (%)**
Primary	33	1	3%
Metastatic			
Lymph Node	27	7	26%
Skin/Subcutaneous	19	6	32%
Visceral	8	4	50%
Primary biopsy from metastatic patients	6	1	17%
Total	60	18	30%

### NEP protein expression in metastatic melanoma: correlation with survival endpoints

There were no statistically significant differences in survival endpoints based on NEP protein expression, however there was a nonsignificant trend towards shorter survival among patients overexpressing NEP. Median overall survival time was 2.5 years shorter for metastatic melanoma patients who overexpressed NEP compared to metastatic melanoma patients not overexpressing NEP [10.2 years (95% CI 4.7–15.7 years) versus 12.7 years (95% CI 7.1–18.3 years) respectively, p = 0.74 by log-rank test]. Patients with metastatic melanoma demonstrating NEP overexpression also had a 2.2 year shorter median disease-free survival time than metastatic melanoma patients not overexpressing NEP [4.9 years (95% CI 1.3–10.1 years) vs. 7.1 years (95% CI 2.2–10.3 years), respectively, p = 0.74]. Finally, two-year post-recurrence survival rates were 67% and 64% for subjects who overexpressed NEP versus those who did not overexpress NEP, respectively (p = 0.89 by log-rank test).

### NEP protein expression correlated with NEP transcript upregulation

**Figure 4:**
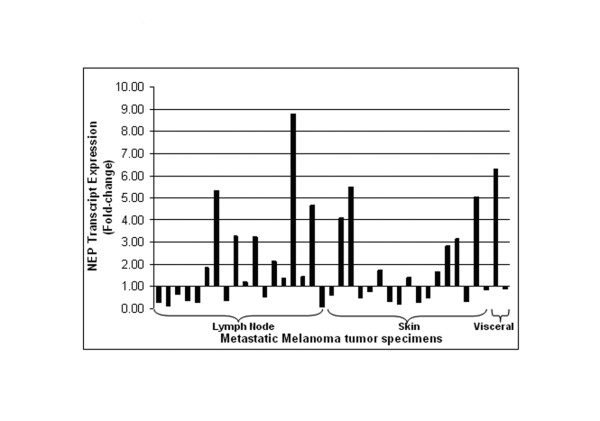
**NEP transcript expression analysis**. Results of gene transcript expression analysis of NEP in 37 metastatic melanoma samples, expressed as a ratio of NEP expression in normal lymph node tissue. Twelve of 37 samples show at least 2-fold increased gene expression.

RNA transcript levels of metastatic tumors were determined using Affymetrix U133Plus2.0 GeneChips and correlated with immunohistochemical analysis of the same tumor tissues. Twelve of 37 (32%) metastatic tumors (6 of 18 lymph node, 5 of 17 skin, and 1 of 2 visceral) had at least two-fold increase of NEP transcripts in comparison to normal lymph node tissue (range 0.09–8.77-fold, Figure 4). Of the 37 metastatic melanoma tumors tested for NEP transcript expression, 25 were also examined for NEP protein expression using immunohistochemistry, including 12 lymph node, 11 subcutaneous and 2 visceral metastases. Table 2 shows the transcript and protein expression for these 25 tumor specimens. Correlation of NEP transcript and protein levels revealed that there was a statistically significant association between increased NEP transcript and NEP protein overexpression, in which all (8/8) of tumors that had greater than 2-fold (range 2.16–8.77–fold) increase in NEP transcripts displayed protein overexpression, whereas only 12% (2/17) of tumors with normal or decreased transcript levels overexpressed NEP (p < 0.0001 by Fisher's exact test). Analysis of these data using the absolute call values of the genechip data revealed similar results (data not shown).

**Table 2:** Correlation between NEP Transcript and Protein expression in 25 melanoma specimens

**Specimen**	**Transcript fold change**	**Protein Expression**
1	8.77	Diffuse
2	6.31	Moderate
3	5.05	Diffuse
4	4.66	Diffuse
5	4.08	Diffuse
6	3.29	Diffuse
7	3.17	Diffuse
8	2.16	Diffuse
9	1.86	Focal
10	1.74	Negative
11	1.65	Negative
12	1.43	Negative
13	1.20	Focal
14	0.89	Diffuse
15	0.77	Focal
16	0.65	Focal
17	0.51	Focal
18	0.49	Focal
19	0.48	Negative
20	0.38	Focal
21	0.37	Negative
22	0.34	Focal
23	0.30	Negative
24	0.28	Diffuse
25	0.27	Negative

### Identification of genes that co-express with NEP

To identify genes/probes that co-express with NEP, we analyzed the geneprobe data from the 25 specimens for which we had both transcript and protein expression data. Among the top 100 gene probes in overall ranks, 41 probes from 38 genes (besides two NEP probes) had their t-test p-values less than 0.005 and were considered co-expressing with NEP. Within these selected genes, 33 were positively correlated with NEP expression (Table 3) and 5 were negatively correlated with the NEP expression (Table 4).

**Table 3:** Genes that positively correlate with NEP

**Gene Probe**	**Gene Symbol**	corA*	corB**	p-value***	**Gene Name**
208469_s_at	EGFL8	0.819	0.852	0.00006	EGF-like-domain, multiple 8
209826_at	PPT2	0.729	0.861	0.00008	palmitoyl-protein thioesterase 2
226270_at	EXOC2	0.659	0.751	0.00006	exocyst complex component 2
201537_s_at	DUSP3	0.632	0.79	0.00006	dual specificity phosphatase 3 (vaccinia virus phosphatase VH1-related)
223057_s_at	XPO5	0.665	0.706	0.00018	exportin 5
209238_at	STX3A	0.729	0.747	0.00048	syntaxin 3A
200824_at	GSTP1	0.729	0.765	0.00086	glutathione S-transferase pi
208899_x_at	ATP6V1D	0.618	0.673	0.00035	ATPase, H+ transporting, lysosomal 34 kDa, V1 subunit D
223113_at	HSPC196	0.718	0.765	0.00135	NA
209490_s_at	PPT2	0.514	0.737	0.00012	palmitoyl-protein thioesterase 2
200692_s_at	HSPA9B	0.557	0.667	0.00035	heat shock 70 kDa protein 9B (mortalin-2)
223056_s_at	XPO5	0.527	0.738	0.00048	exportin 5
202475_at	NIFIE14	0.514	0.781	0.00035	NA
227124_at	NA	0.665	0.753	0.00147	NA
203723_at	ITPKB	0.718	0.646	0.00086	inositol 1,4,5-trisphosphate 3-kinase B
200924_s_at	SLC3A2	0.527	0.697	0.0009	solute carrier family 3 (activators of dibasic and neutral amino acid transport), member 2
211968_s_at	HSP90AA1	0.43	0.831	0.00006	heat shock protein 90 kDa alpha (cytosolic), class A member 1
219526_at	C14orf169	0.49	0.615	0.00035	chromosome 14 open reading frame 169
218465_at	TMEM33	0.729	0.697	0.00307	transmembrane protein 33
200691_s_at	HSPA9B	0.632	0.653	0.00243	heat shock 70 kDa protein 9B (mortalin-2)
212793_at	DAAM2	0.618	0.566	0.00064	dishevelled associated activator of morphogenesis 2
201089_at	ATP6V1B2	0.618	0.601	0.0019	ATPase, H+ transporting, lysosomal 56/58 kDa, V1 subunit B2
218305_at	IPO4	0.43	0.683	0.00035	importin 4
32836_at	AGPAT1	0.618	0.56	0.00113	1-acylglycerol-3-phosphate O-acyltransferase 1 (lysophosphatidic acid acyltransferase, alpha)
201195_s_at	SLC7A5	0.43	0.642	0.00048	solute carrier family 7 (cationic amino acid transporter, y+ system), member 5
202088_at	SLC39A6	0.418	0.825	0.00064	solute carrier family 39 (zinc transporter), member 6
205512_s_at	PDCD8	0.527	0.604	0.00307	programmed cell death 8 (apoptosis-inducing factor)
1554679_a_at	LAPTM4B	0.665	0.545	0.0019	lysosomal associated protein transmembrane 4 beta
226101_at	PRKCE	0.514	0.542	0.00064	protein kinase C, epsilon
214359_s_at	HSP90AB1	0.402	0.651	0.00035	heat shock protein 90 kDa alpha (cytosolic), class B member 1
239406_at	ZNF193	0.49	0.642	0.00387	zinc finger protein 193
204015_s_at	DUSP4	0.665	0.6	0.00483	dual specificity phosphatase 4
201771_at	SCAMP3	0.43	0.583	0.00147	secretory carrier membrane protein 3
203914_x_at	HPGD	0.618	0.561	0.00483	hydroxyprostaglandin dehydrogenase 15-(NAD)
211373_s_at	PSEN2	0.43	0.542	0.0019	presenilin 2 (Alzheimer disease 4)
220272_at	BNC2	0.659	0.575	0.00736	basonuclin 2

*corA = calculated correlation dichotomized based on ratio to lymph node

**corB = Pearson correlation

***p-value for w-stat: p-value for Wilcoxan rank-sum test

**Table 4:** Genes that negatively correlate with NEP

**Gene Probe**	**Gene Symbol**	corA*	corB**	p-value***	**Gene Name**
203865_s_at	ADARB1	-0.542	-0.566	0.00483	adenosine deaminase, RNA-specific, B1 (RED1 homolog rat)
203324_s_at	CAV2	-0.601	-0.534	0.0043	caveolin 2
226899_at	UNC5B	-0.659	-0.479	0.00064	unc-5 homolog B (C. elegans)
227162_at	ZBTB26	-0.385	-0.541	0.00008	zinc finger and BTB domain containing 26
225923_at	VAPB	-0.487	-0.518	0.00307	VAMP (vesicle-associated membrane protein)-associated protein B and C

*corA = calculated correlation dichotomized based on ratio to lymph node

**corB = Pearson correlation

***p-value for w-stat: p-value for Wilcoxan rank-sum test

## Discussion

We investigated the clinical relevance of altered NEP expression in a well-characterized cohort of melanoma patients. We chose to examine NEP expression in melanoma since there is growing evidence that upregulation of its expression may relate to tumor progression. Furthermore, the availability of NEP inhibitors [14,15] makes NEP a potential therapeutic target. We first screened several human melanoma cell lines to explore *in-vitro *the frequency of the NEP protein expression before utilizing more precious clinical specimens linked to extended follow up information. We also examined increased transcription as a possible mechanism of NEP protein overespression. Our study reveals that NEP overexpression is common in patients with metastatic melanoma and is related to increased transcription. It also suggests an association with visceral spread of disease that requires validation in a larger dataset.

While our initial plan was to explore the correlation between NEP overexpression in primary melanoma and clinicopathological parameters of poor outcome and survival, we limited our study to only 33 primary tumors due the very low frequency of altered NEP expression in this particular clinical setting. Thirty-two of the 33 cases showed undetectable NEP expression. The only primary tumor that showed NEP overexpression (diffuse expression) was an acral melanoma, which has already been established as having distinct biological and genetic underpinnings compared to non-acral cutaneous melanoma [21]. We decided, therefore, to conserve the utilization of tissues from primary cases and limited our report to 33 cases. This low rate of overexpression seen in primary lesions is lower than two studies that reported a frequency of 20%[22,23]. The discordance is most likely attributable to the fact that previous studies included a greater number of thick primary tumors [22]. The relatively small number of thick melanomas in our study cohort reflects the nationwide trend towards thinner lesions at the time of diagnosis over the last few decades [24]. In addition, we used a rigorous cutoff point of 20% in determining overexpression, which is supported by the significant correlation between ≥2 fold increase in NEP transcripts and overexpression of its protein using immunohistochemistry. Previous studies defined NEP overexpression as any expression more than 1% or 10% staining [22,23]. We also found that there is a trend towards increased NEP expression in visceral metastases, suggesting that NEP overexpression may be related to certain biological behaviors of tumor spread. However, our ability to better define this relationship is limited by the availability of tumor tissue from visceral metastases, as most patients do not undergo biopsies of these tumors as part of their standard clinical care. Thus most available metastatic samples are skin and lymph node metastases. Nevertheless, the observation that NEP overexpression is correlated with visceral metastases has been previously reported in colorectal cancer [7]. In that study, NEP overexpression was an independent predictor of liver metastasis (n = 505). These data suggest that increased NEP expression relates to hematologic metastasis of a subset of solid tumors.

We next examined whether NEP expression correlated with patient survival. We analyzed three measures of survival, including overall survival time from initial melanoma diagnosis, time from initial diagnosis to first recurrence, and survival time after first recurrence. No significant differences were detected for any of the survival measurements. Previously published work, based on findings of only two patients, suggested that patients with NEP-positive metastases may have shorter survival times than those with NEP-negative metastases [23]. A larger sample size with extended follow-up is needed to validate these findings. In addition, the inclusion of more visceral metastases might be considered.

We found a strong correlation between increased NEP transcription and its protein overexpression, suggesting that the protein accumulation is secondary to increased production (as is the case with other oncogenes such as epidermal growth factor receptor and cyclin D1 in lung and esophageal cancer, respectively) rather than an increase in protein half-life or decrease in protein degradation [25-27]. Of note, our *in vitro *screening was not meant to appropriately address the functional mechanism of NEP regulation; it only served as a pilot experiment to explore the frequency of NEP protein overexpression in melanoma and the role of transcription in its up-regulation before we embarked upon the utilization of more precious human tissues.

We analyzed the microarray data to identify genes that co-expressed with NEP. Interestingly, we found 33 genes that positively correlated with NEP, and 5 genes that negatively correlated with NEP. The genes that positively correlated with NEP expression included those in the MAP kinase pathway (n = 4), antigen processing and presentation (n = 4), apoptosis (n = 1), and WNT signaling pathway (n = 1) [16]. Specifically, a number of the genes identified have been found to be overexpressed in melanoma and other cancers, including glutathione S transferase pi, which is overexpressed in melanoma, and is thought to play a role in melanoma drug resistance [28], and lysosomal associated protein transmembrane 4 beta, which is overexpressed in numerous solid tumors such as hepatocellular carcinoma, lung cancer and colon cancer, and has been shown to increase proliferation rates and anchorage independent growth in tumor cell lines [29,30]. Additionally, protein kinase C epsilon is expressed in many human melanoma cell lines, and has been hypothesized to be advantageous for in vivo growth of melanoma cells [31,32].

The 5 genes that were negatively correlated with NEP expression included genes involved in focal adhesion (n = 1) and the notch signaling pathway (n = 1) [16]. One of these genes, Unc-5 homolog B, has been shown to be down-regulated in multiple cancers, and appears to function as a tumor suppressor by mediating p53-dependent apoptosis [33,34]. Another gene that negatively correlated with NEP expression was caveolin 2, the expression of which is decreased in breast cancer tissue when compared to normal tissue and is correlated with hormone receptor status [35]. Overall, NEP expression appears to correlate with a number of important pathways in tumor growth and development, and further investigations are needed to determine whether NEP plays a causal role in these relationships, or is upregulated as a secondary effect of tumor growth.

In conclusion, we demonstrated that NEP expression relates to the progression of melanoma from primary to metastatic disease, and may highlight biological differences in the pattern of spread of metastatic disease. More functional investigations are needed to further understand the mechanism and role of NEP expression in melanoma progression.

## Competing interests

The author(s) declare that they have no competing interests.

## Authors' contributions

EV participated in the study design, analyzed the histology and immunohistochemistry and participated in manuscript drafting. MY participated in correlating clinical and molecular data and participated in manuscript writing. AP, RB and RS enrolled patients into the study and participated in writing the manuscript. DB, DO and YY carried out the gene expression analysis. JS coordinated data collection. PC, XZ and MM carried out statistical analysis. DN participated in study design, interpretation of the data and manuscript writing. LL participated in data analysis. NB helped conceive of the study and coordinated the gene expression analysis. DP contributed to the study design and drafting of the manuscript. IO designed the study, and led the data interpretation and manuscript writing.
